# Double-perovskite magnetic La_2_NiMnO_6_ nanoparticles for adsorption of bovine serum albumin applications

**DOI:** 10.1186/1556-276X-8-207

**Published:** 2013-05-02

**Authors:** Zhi-Yong Wu, Cai-Bin Ma, Xin-Gui Tang, Rui Li, Qiu-Xiang Liu, Bao-Tian Chen

**Affiliations:** 1 Department of Traditional Chinese Medicine of Nanfang Hospital, Southern Medical University, Guangzhou 510515, People’s Republic of China; 2 School of Physics and Optoelectric Engineering, Guangzhou Higher Education Mega Center, Guangdong University of Technology, Guangzhou 510006, People’s Republic of China

**Keywords:** Chemical co-precipitation, LNMO, Magnetic property, Nanoparticles, Adsorption, Bovine serum albumin protein

## Abstract

Double-perovskite La_2_NiMnO_6 _(LNMO) nanoparticles were synthesized by co-precipitation process, and the adsorption of bovine serum albumin (BSA) protein on these nanoparticles was carried out. The powder samples were annealed at 750, 850, 950, and 1,050°C, respectively. X-ray diffraction (XRD) results reveal that there are double perovskites and exhibit mixed orientations, without any impurity phases. Transmission electron microscopy results as well as the XRD estimate results show that the crystalline size is about 34 to 40 nm. The adsorption of BSA on the magnetic nanoparticles was analyzed using a UV spectrophotometer at room temperature. The results show that the as-prepared LNMO nanoparticles display a good adsorbing ability for BSA, and the nanoparticle sintered at 850°C has the highest value of 219.6 mg/g, which is much higher than others.

## Background

Magnetic nanoparticles are commercially important materials as a consequence of their stability and striking magnetic property [1] and are applied widely in biological and medical areas, such as bioseparation [2], drug and gene delivery [3], quantitative immunoassay [4], and hyperthermia [5].

Recently, magnetic nanoparticles, such as CoFe_2_O_4_, MnFe_2_O_4_, Fe_2_O_3_, Fe_3_O_4_, and Fe [6-10], have been studied mostly for biomedical applications, but the application of double-perovskite La_2_NiMnO_6 _nanoparticles in biomedical has not been reported. Double-perovskite La_2_NiMnO_6 _is a ferromagnetic material and attractive due to its impressive properties. In order to be applied in biological and medical fields, La_2_NiMnO_6 _nanoparticles should be monodispersed to bind biomolecules. Proteins are relatively large biomolecules and usually have a tendency to accumulate at the interface between aqueous solutions and solid surfaces [11-15]. Protein adsorption to surfaces is important in many disciplines, including biomedical engineering, biotechnology, and environmental science.

Many works were used to research the magnetic characteristics of double-perovskite nanoparticles. There has been no report about the application of these nanoparticles in biomedicine. Our experiments show that different annealing temperatures can affect the adsorbing ability for bovine serum albumin (BSA). In this paper, we report that the monodispersed La_2_NiMnO_6 _nanoparticles were synthesized by co-precipitation, and the magnetic properties and adsorption characteristics of bovine serum albumin for these nanoparticles were analyzed.

## Methods

The La_2_NiMnO_6 _(LNMO) nanocomposites were synthesized by co-precipitation, using La(NO_3_)_3_·5H_2_O(99.5%), Ni(CH_3_COO)_2_·4H_2_O (98%), and Mn(CH_3_COO)_4_·4H_2_O(99%) as starting raw materials [16]. The raw powders were dissolved in deionized water in required stoichiometric proportions. The solutions were then poured together into a beaker and stirred in a magnetic blender at 80°C. After 2 h, aqueous ammonia solution was added to the container until a brown suspension took shape at a pH of approximately 8.5 [17]. After stirring for about 30 min, the suspension was ball-milled for 24 h with ethanol as a milling medium in order to mix the reactants well enough and then dried in a cabinet dryer at 80°C overnight to obtain the precursor samples. The dried powders were finally annealed in nitrogen atmosphere for 2 h at different temperatures in the range of 750°C~1,050°C.

The crystalline phase of LNMO nanocomposites was identified using the X-ray diffraction (XRD) technique. The X-ray diffractogram of all the samples from 10° to 70° at a scanning step of 0.02°/s was recorded using a Rigaku X-ray diffractometer (Rigaku Corporation, Tokyo, Japan) with Cu Kα radiation (*λ* = 1.54056 Ǻ ). The magnetic properties were measured using a vibrating sample magnetometer (PPMS-9, Quantum Design, Inc., San Diego, CA, USA) at room temperature under a maximum field of 30 kOe. The structural defects in the LNMO materials were investigated using a JEOL 4000EX high-resolution transmission electron microscope (HRTEM; JEOL Ltd., Tokyo, Japan) operated at 400 kV. The adsorption of BSA protein on nanoparticles was analyzed with a UV spectrophotometer (UV-2401 PC, Shimadzu Corporation, Kyoto, Japan) at room temperature. The aqueous solution with a pH of about 7.4 contained 1.000 mg/ml BSA (purity >99%) before the adsorption, and for each measurement, 3.00 to 12.00 mg of La(Ni_0.5_Mn_0.5_)O_3 _nanoparticles was used as the adsorbent. The adsorbent was stirred ultrasonically in the BSA solution for 1 h at room temperature, which was put in static precipitation condition after 12 h to be measured.

## Results and discussion

**Figure 1:**
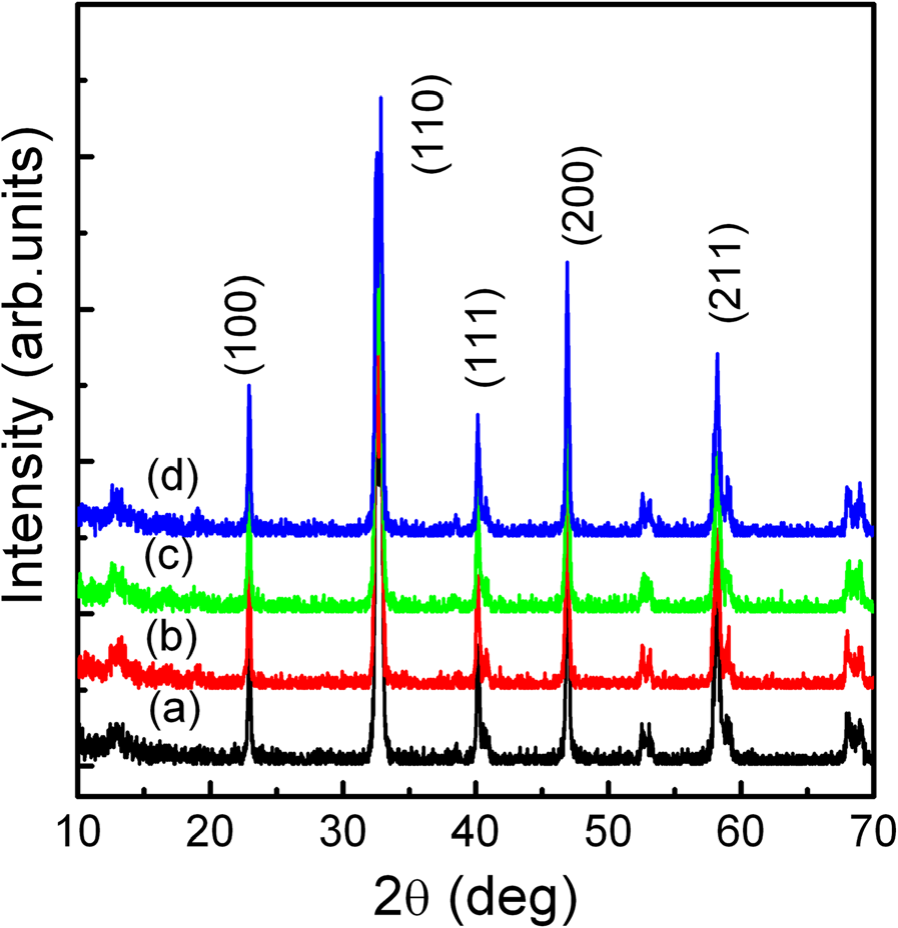
**XRD patterns of LNMO nanoparticles annealed at different temperatures for 2 h.** (**a**) 750°C, (**b**) 850°C, (**c**) 950°C, and (**d**) 1,050°C.

Figure 1 presents the XRD patterns for the whole samples with temperatures ranging from 750°C to 1,050°C. All of the diffraction peaks are identified and indexed according to the standard diffraction pattern data of LNMO powders. As seen from the scan (Figure 1), the LNMO nanoparticles have formed a pure perovskite and exhibit random orientation [18,19]. The lattice constants of LNMO are *a* = 5.467 Ǻ, *b* = 5.510 Ǻ, *c* = 7.751 Ǻ, and *β* = 91.12*°*. It is also observed that the diffraction peaks become narrower and sharper with the increase of annealing temperature, indicating an augmentation of the crystallite size, which has been confirmed by the crystallite size calculation using the Scherrer formula at (100) and (200) peaks:

(1) $$D=\frac{0.9\lambda }{\beta \cos\theta },$$

where *D* is the crystallite size, *λ* is the wavelength of Cu Kα, *β* is the full width at half maximum of the diffraction peaks, and *θ* is the Bragg angle. The calculated crystallite sizes are shown in Table 1. As the annealing temperature increases from 750°C to 1,050°C, the grain sizes of the nanocrystallites increase from 33.9 to 39.6 nm.

**Table 1:** **Average grain size and magnetic and BSA adsorption properties of La(Ni**_**0.5**_**Mn**_**0.5**_**)O**_**3 **_**nanoparticles**

**Annealing temperature (°C)**	**Grain size (nm)**	***M***_**S **_**(×10**^**−3 **^**emu/g)**	***H***_**C **_**(Oe)**	**Nanoparticle mass (mg)**		**BSA adsorbed (mg/g)**	
				**a**	**b**	**a**	**b**
750	33.9	1.97	37.5	5.5	7.8	51.00	36.84
850	36.5	3.1	19.9	6.5	8.2	189.35	219.61
950	37.9	1.97	42.3	5.4	7.2	51.94	30.24
1,050	39.6	3.79	39.9	7.1	7.4	27.68	33.04

The nanoparticles were annealed at different temperatures for 2 h.

LaMnO_3_ is an ABO_3 _perovskite ferromagnetic material. The ionic radius of Ni^3+^ (62 pm) is smaller than that of Mn^3+ ^(66 pm). Therefore, an inhomogeneous distribution results at the B site of the structure. A cationic disorder induced by B-site substitution is always regarded as the main derivation of crystalline growth. On the other hand, LaNiO_3 _is a paramagnetic material; the La ion locates at the central equilibrium position of the LaNiO_3_ lattice. In this case, the macrodomain in LaMnO_3 _could be divided into the microdomains which probably cause the crystalline growth. Because the domain size relates to the grain sizes, the grain size increases slowly when the annealing temperature increases.

**Figure 2:**
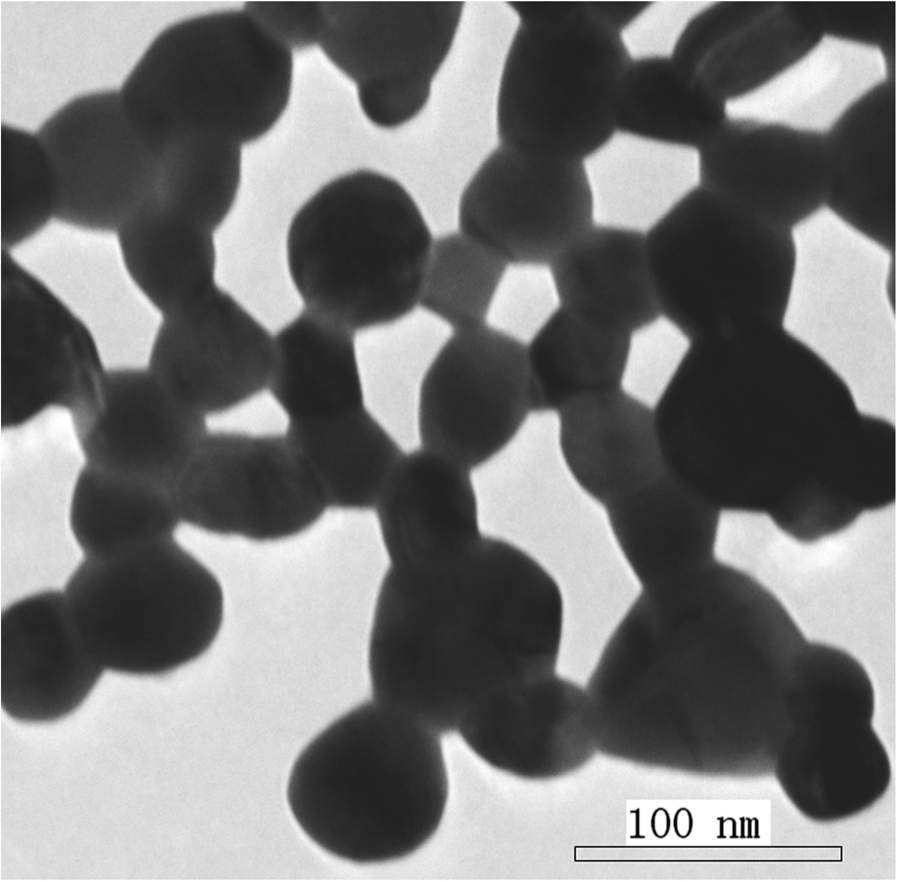
**The HRTEM morphology of the LNMO sample annealing at 750°C for 2 h.**

Figure 2 shows the TEM morphology of the obtained LNMO nanoparticles. It can be observed from the TEM morphology and XRD analysis that the LNMO nanoparticles form a group of cluster phenomenon and that the average grain size is about 40 nm.

**Figure 3:**
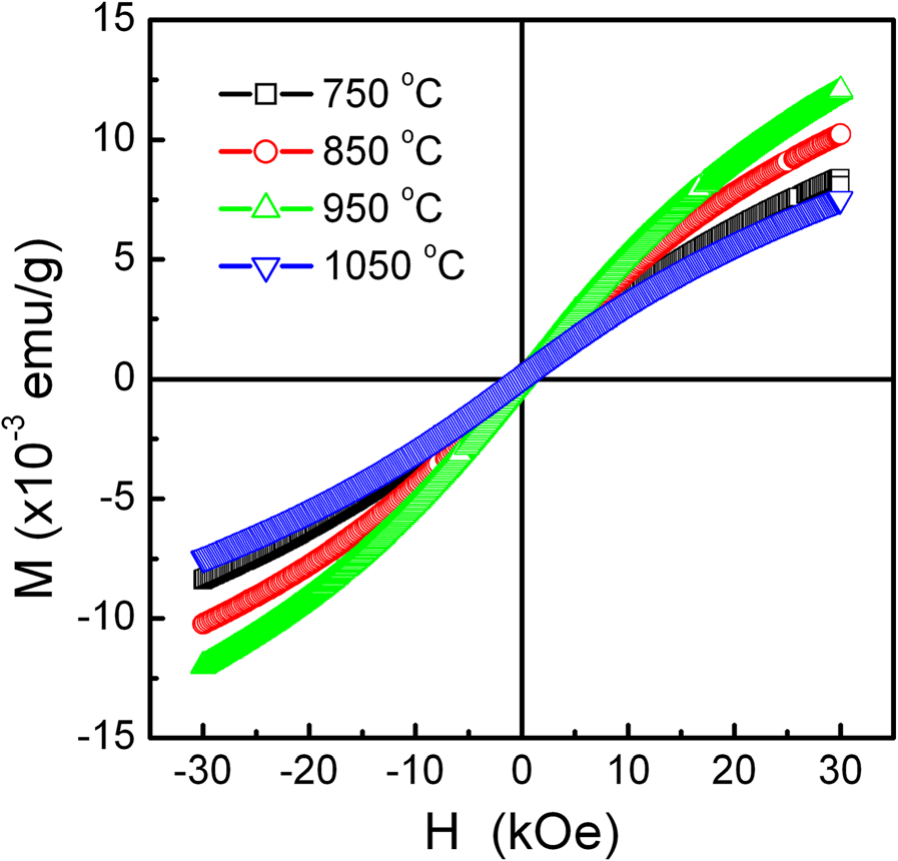
Field-dependent magnetization hysteresis of LNMO samples annealing at various temperatures.

The magnetic hysteresis loops of the samples annealed at 750°C, 850°C, 950°C, and 1,050°C are shown in Figure 3. It is seen that the whole magnetization curves are not saturated at a maximum external field of 30 kOe and that the hysteresis curves for all samples are ‘S’ shaped with very low coercivity (*H*_C _< 45 Oe); both of which are characteristics of the superparamagnetism as reported in [18-20]. Superparamagnetic particles could be fit to a simple Langevin theory *M*(*H*)/*M*_*S*_ = *L*(*x*), where *M*(*H*) is the magnetization for an applied field *H*, and *M*_S _represents the saturation magnetization. Thus, by applying the curves to the Langevin formula, we should be able to approximately determine *M*_S_[20,21]. In the Langevin function, *L*(*x*) = coth *x* − 1/*x*, where *x* = *μH*/*k*_*B*_*T*, *μ* is the uncompensated magnetic moment, *k*_*B *_stands for Boltzmann’s constant, and *T* represents the absolute temperature. For high fields, it gives 1 − *k*_*B*_*T*/*μH* for the form of the approach to saturation. Consequently, we may come to a conclusion: *M*(*H*) = *M*_*S*_(1 − *a*/*H*), where the term *α* is used to replace some constants mentioned above in order to simplify the formula.

The UV spectra of BSA at 280 nm were recorded to investigate the BSA binding capacity of the LNMO nanoadsorbents; 1-mg nanoadsorbents’ adsorptive capacity of BSA is calculated by Equation 2 [22]:

(2) $$\eta =\frac{m_{\mathrm{BSA}}\left(A_{\mathrm{BSA}}-A_{\text{mag}}\right)}{m_{\text{mag}}A_{\mathrm{BSA}}},$$

where *η* indicates the amount of 1-mg nanoadsorbents (mg/g) in the adsorbed BSA, *m*_BSA _is the total weight of BSA (mg), *m*_mag _is the dry weight of nanopowders used to bind BSA (mg), *A*_BSA _points to the UV absorbance value of the blank BSA solution, and *A*_mag _refers to the UV absorbance value of the supernatant after adsorption.

### Adsorption of bovine serum albumin on LNMO nanoparticles

BSA is a globular protein with the approximate shape of a prolate spheroid with dimensions of 4 nm × 4 nm × 14 nm [23]. Table 1 shows BSA adsorption on the LNMO nanoparticles. From Table 1, it can be seen that the LNMO nanoparticles exhibit a good absorbing characteristic for BSA protein. The BSA adsorption capability on the LNMO nanoparticles is influenced possibly by their grain size, specific surface area, magnetic properties, interface structure, the electrostatic attraction between BSA and magnetic nanoparticles, etc., which are related to the preparation process. The LNMO nanoparticles annealed at 850°C show the highest BSA adsorption at around 219.6 mg/g. On this circumstance, the volume of the aqueous BSA solution after adsorption was increased to about 3 ml. The LNMO nanoparticles annealed at 850°C showed the lowest coercive field (19.9 Oe, see Table 1) and have the highest BSA adsorption at around 219.6 mg/g; the main reason is based on the critical grain size of LNMO nanoparticles for BSA adsorption.

The reason for this is not clear, and it needs a further systematic study. In fact, up to now, protein adsorption mechanism on nanoparticles is not fully understood although it has been intensively investigated by researchers [24,25].

## Conclusions

In conclusion, La(Ni_0.5_Mn_0.5_)O_3 _(LNMO) nanoparticles have been successfully prepared using the chemical co-precipitation process. The grain size and magnetic properties of the LNMO nanoparticles are largely influenced by annealing temperature. As the annealing temperature increases from 750°C to 1,050°C, the average grain size increases from about 33.9 to 39.6 nm, respectively. The saturation magnetization increases from about 35.95 to 67.19 emu/g; However, as the annealing temperature increases from 950°C to 1,050°C, the average grain size decreases from about 37.9 to 39.6 nm, and the saturation magnetization decreases from about 1.97×10^-3 ^to 3.79×10^-3 ^emu/g. On the other hand, the coercivity initially increases, reaching a maximum value of 42.3 Oe when the average grain size is about 37.9 nm at 950°C, and then reduces. The LNMO nanoparticles showed good adsorption performance in bovine serum albumin protein, and the preliminary optimized adsorption is obtained for the LNMO nanoparticles annealed at 850°C. These LNMO nanoparticles are a potential carrier for large biomolecules, which will be widely used in the biomedical field.

## Competing interests

The authors declare that they have no competing interests.

## Authors’ contributions

ZYW, CBM, and RL carried out the sample preparation, participated on its analysis, performed all the analyses, and wrote the paper. XGT and QXL helped perform the XRD and FM analyses. XGT and TBC guided the study and participated in the paper correction. All authors read and approved the final manuscript.
